# The effects of vitamin D supplementation on frailty in older adults at risk for falls

**DOI:** 10.1186/s12877-022-02888-w

**Published:** 2022-04-10

**Authors:** Yurun Cai, Amal A. Wanigatunga, Christine M. Mitchell, Jacek K. Urbanek, Edgar R. Miller, Stephen P. Juraschek, Erin D. Michos, Rita R. Kalyani, David L. Roth, Lawrence J. Appel, Jennifer A. Schrack

**Affiliations:** 1 Department of Epidemiology, Johns Hopkins Bloomberg School of Public Health, Baltimore, MD USA; 2 Department of Community and Health Systems, University of Pittsburgh School of Nursing, Pittsburgh, PA USA; 3 Center On Aging and Health, Johns Hopkins University, Baltimore, MD USA; 4 Welch Center for Prevention, Epidemiology, and Clinical Research, Johns Hopkins University and Medical Institutions, Baltimore, MD USA; 5 Division of Geriatric Medicine and Gerontology, Johns Hopkins School of Medicine, Baltimore, MD USA; 6 Division of General Internal Medicine, Johns Hopkins School of Medicine, Baltimore, MD USA; 7 Division of General Medicine, Beth Israel Deaconess Medical Center, Harvard Medical School, Boston, MA USA; 8 Division of Cardiology, Johns Hopkins School of Medicine, Baltimore, MD USA; 9 Division of Endocrinology, Johns Hopkins School of Medicine, Diabetes, & Metabolism, Baltimore, MD USA

**Keywords:** Frailty, Nutrition supplementation, Vitamin D3, Randomized controlled trial

## Abstract

**Background:**

Low serum 25-hydroxyvitamin D [25(OH)D] level is associated with a greater risk of frailty, but the effects of daily vitamin D supplementation on frailty are uncertain. This secondary analysis aimed to examine the effects of vitamin D supplementation on frailty using data from the Study To Understand Fall Reduction and Vitamin D in You (STURDY).

**Methods:**

The STURDY trial, a two-stage Bayesian, response-adaptive, randomized controlled trial, enrolled 688 community-dwelling adults aged ≥ 70 years with a low serum 25(OH)D level (10–29 ng/mL) and elevated fall risk. Participants were initially randomized to 200 IU/d (control dose; n = 339) or a higher dose (1000 IU/d, 2000 IU/d, or 4000 IU/d; n = 349) of vitamin D3. Once the 1000 IU/d was selected as the best higher dose, other higher dose groups were reassigned to the 1000 IU/d group and new enrollees were randomized 1:1 to 1000 IU/d or control group. Data were collected at baseline, 3, 12, and 24 months. Frailty phenotype was based on number of the following conditions: unintentional weight loss, exhaustion, slowness, low activity, and weakness (≥ 3 conditions as frail, 1 or 2 as pre-frail, and 0 as robust). Cox proportional hazard models estimated the risk of developing frailty, or improving or worsening frailty status at follow-up. All models were adjusted for demographics, health conditions, and further stratified by baseline serum 25(OH)D level (insufficiency (20–29 ng/mL) vs. deficiency (10–19 ng/mL)).

**Results:**

Among 687 participants (mean age 77.1 ± 5.4, 44% women) with frailty assessment at baseline, 208 (30%) were robust, 402 (59%) were pre-frail, and 77 (11%) were frail. Overall, there was no significant difference in risk of frailty outcomes comparing the pooled higher doses (PHD; ≥ 1000 IU/d) vs. 200 IU/d. When comparing each higher dose vs. 200 IU/d, the 2000 IU/d group had nearly double the risk of worsening frailty status (HR = 1.89, 95% CI: 1.13–3.16), while the 4000 IU/d group had a lower risk of developing frailty (HR = 0.22, 95% CI: 0.05–0.97). There were no significant associations between vitamin D doses and frailty status in the analyses stratified by baseline serum 25(OH)D level.

**Conclusions:**

High dose vitamin D supplementation did not prevent frailty. Significant subgroup findings might be the results of type 1 error.

**Trial registration:**

ClinicalTrials.gov: NCT02166333.

**Supplementary Information:**

The online version contains supplementary material available at 10.1186/s12877-022-02888-w.

## Introduction

Frailty, as a phenotype of age-associated vulnerability, has been identified as a clinical state/syndrome of decreased reserve and resistance to stressors [1, 2]. This compromised state is attributable to age-related declines across multiple physiologic systems that may be clinically recognizable through five key clinical signs and symptoms: unintentional weight loss, muscle weakness, exhaustion, slow gait speed, and low physical activity [1, 2]. Frailty is more prevalent in older age and is associated with adverse health outcomes including falls, disability, hospitalization, and mortality [3–5]. Identifying effective interventions to reduce the risk of frailty may also decrease the risk of these adverse health outcomes and help maintain functional independence.

Previous clinical trials and observational studies suggest that vitamin D supplementation may improve muscle strength and mobility, with effects that are stronger among adults aged $\ge$65 years or those with vitamin D deficiency [6–10]. A number of prospective cohort studies have demonstrated an association between low serum 25-hydroxyvitamin D (25(OH)D) levels and incident frailty [11–14]; however, it is unknown that whether vitamin D supplementation is effective at reducing risk of frailty in older adults [15].

In this secondary analysis of data from the STURDY (Study To Understand Fall Reduction and Vitamin D in You) clinical trial, we examined the effects of vitamin D supplementation on frailty status in community-dwelling older adults aged 70 and older. The primary aim of the STURDY trial was to examine whether high-dose vitamin D supplementation would reduce the risk for falls [16]. Although the primary trial findings indicated that vitamin D supplementation did not prevent falls, the effects of vitamin D supplementation on the risk of frailty in the STURDY cohort have not been previously reported. We hypothesized that participants randomized to higher doses of supplemental vitamin D (≥ 1000 IU/d) would have a lower risk of incident frailty over 24 months of follow-up compared to the control dose (200 IU/d).

## Methods

The STURDY trial was a two-stage, Bayesian response-adaptive dose-finding and seamless confirmatory randomized trial. The rationale and design of STURDY and the primary outcome results have been published [16, 17]. Briefly, the study recruited community-dwelling older persons with low serum 25(OH)D and high risk for falls. Participants were initially randomized to daily doses of a single pill containing either 200 IU (control), 1000 IU, 2000 IU, or 4000 IU of vitamin D3. At the end of the dose-finding stage, the 1000 IU/d dose was selected as the best higher dose. Other higher dose groups (2000 IU/d and 4000 IU/d) were then switched to the best dose group, and new enrollees were randomized 1:1 to 1000 IU/d or control group. The data and safety monitoring board (DSMB) recommended termination of the trial on 2/8/2019 after review of trial data indicated sufficient power to address the primary research question of vitamin D_3_supplementation and falls. Randomization ended on 2/11/2019, and data collection ended on 5/31/2019. The trial protocol was approved by the Johns Hopkins University institutional review board and published in the appendix of the main results paper [16]. Written informed consent was obtained from all participants. All methods were performed in accordance with the relevant guidelines and regulations.

### Participants

Community-dwelling older adults aged ≥ 70 years with elevated fall risk and serum 25(OH)D level of 10–29 ng/mL were eligible to participate in the trial. Elevated fall risk was defined by self-report of at least one of the following: ≥ 1 injurious fall or ≥ 2 falls in the past year regardless of injury, fear of falling due to balance or walking problems, difficulty maintaining balance, or use of an assistive device when walking. Major exclusion criteria included cognitive impairment and use of personal vitamin D supplement > 1000 IU/day or calcium supplements > 1200 mg/d.

### Treatment

Eligible participants were randomized to four cholecalciferol (vitamin D3) dose groups: 200 IU/d (control), 1000 IU/d, 2000 IU/d, or 4000 IU/d. The rationale and safety for these four vitamin D3 dose levels are explained elsewhere [16, 17]. All pills containing different doses had identical appearance and were manufactured by Continental Vitamin Company (Vernon, CA). Duration of pill-taking and follow-up was 2 years or end of the trial, whichever came first.

### Randomization

Randomization of participants began on 10/30/2015. The assignment probability to the 200 IU/d group was 0.50 throughout the trial. For higher dose non-control groups (1000 IU/d, 2000 IU/d, and 4000 IU/d), each group had equal probability of assignment (0.1667) at the start of the trial. During the dose-finding stage, the probabilities of assignment to non-control doses were adjusted at pre-specified times, beginning after the 100^th^ participant randomized to a non-control dose group achieved 6 months of follow-up. The first adaptation of randomization probabilities occurred on 08/02/2017. The dose-finding stage ended on 3/23/2018 and the 1000 IU/d dose was selected as the best non-control dose, as the lowest fall rates were observed in the 1000 IU/d group. After the dose-finding stage, other higher dose groups (2000 IU/d and 4000 IU/d) were switched to the best dose group (1000 IU/d) and new enrollees were randomized 1:1 to 1000 IU/d or control group. The randomization process ended on 02/11/2019 and data collection ended on 05/31/2019. Study personnel and participants were masked to randomized dose, occurrence of adaptations, and the transition from dose-finding to confirmatory stage.

### Assessments

Participants had clinic visits at baseline and 3, 12, and 24 months after randomization. At each visit, study personnel collected physical measurements including all components of the frailty phenotype and assessed medical events, compliance with supplements, and occurrence of falls.

### Frailty phenotype

Frailty phenotype was defined as having three or more of the following conditions: weight loss (body mass index (BMI) < 18.5 kg/m^2^ or > 5% body weight unintentionally lost in the past year), exhaustion (self-reported tiredness or weakness), slowness (slow 4-m gait speed based on sex- and height-adjusted criteria), low physical activity (sex-adjusted low physical activity energy expenditure per week), and weakness (sex- and BMI-adjusted low grip strength) [18]. Participants without any criteria were classified as robust and those with one or two criteria were classified as pre-frail. Frailty was determined as missing if 3 or more of the 5 components were not assessed.

### Covariates

Age, sex, race/ethnicity, education years, and marital status were self-reported. BMI was calculated from measured weight and height (kg/m^2^). Participants were asked whether a physician ever told them they had any of the following medical conditions: heart disease, high cholesterol, high blood pressure, cancer, stroke, peripheral vascular disease, chronic obstructive pulmonary disease, diabetes, kidney disease, liver disease, connective tissue disease, arthritis, Parkinson’s disease, and multiple sclerosis. The number of comorbidities was summarized. History of falls in the past year (fall or no fall) was included as a covariate.

### Statistical analysis

Sociodemographic characteristics, health conditions, fall history, and frailty status at baseline were compared across vitamin D dose groups. Frequency (percentage) of frailty status at baseline, 3, 12, and 24 months were tabulated by vitamin D dose groups. The number and proportion of participants with improvement, no change, or worsening in frailty status from baseline to each follow-up visit were calculated by vitamin D treatment groups.

Separate Cox proportional hazard models were used to compare the time from randomization to: 1) incident frailty (from robust or pre-frail to frail), 2) improvement in frailty (from frail to pre-frail or robust, or from pre-frail to robust), and 3) worsening of frailty (from robust to pre-frail or frail, or from pre-frail to frail) over follow-up by vitamin D groups. For the analysis with improving frailty status as the outcome, participants who were robust at baseline were excluded. Similarly, for the analysis of developing frailty or worsening frailty status, participants who were frail at baseline were excluded. Additional exploratory analyses were conducted to examine the time to develop each frailty component using Cox proportional hazard models. For analyses in which a frailty component was the outcome, participants who were impaired in the particular frailty component at baseline were excluded. All multivariable models were adjusted for age, sex, race, BMI, comorbidities, baseline serum 25(OH)D level, and fall history. Given evidence that serum 25(OH)D level may moderate the association [6], we further stratified the Cox proportional hazards models by baseline serum 25(OH)D level (20–29 ng/mL defined as vitamin D insufficiency vs 10–19 ng/mL defined as vitamin D deficiency [19]), adjusting for other covariates.

Generalized estimating equations (GEE) models were used to examine the changes in odds of frailty over time by treatment group. Pre-frail and robust participants were collapsed to the non-frail group for the GEE models. Baseline frailty status was adjusted for, and an interaction term for time*treatment was included in the model. Multivariable models were adjusted for demographic and health characteristics. The GEE models were additionally stratified by baseline serum 25(OH)D level.

Consistent with the trial’s design that the confirmatory phase was of principal interest, the primary comparison was between the pooled higher doses (PHD; combined 1000 IU/d, 2000 IU/d, and 4000 IU/d) group and the 200 IU/d group; this comparison allowed use of data from all randomized participants regardless of dose assignment. Sensitivity analyses were conducted to compare participants randomized to 1000 IU/d versus 200 IU/d group (labeled ‘Pure’ analysis). Using data from the burn-in cohort of the dose-finding stage, we compared each higher dose group to 200 IU/d group. The burn-in cohort from the dose-finding phase is an unbiased population for comparison of each higher dose versus control because these participants were randomized prior to the first adaptation of the randomization probabilities. These sensitivity analyses were considered exploratory analyses following the primary analysis.

Two-sided tests with a significance level of 0.05 were used. All analyses were conducted in SAS software version 9.4 (SAS Institute, Inc., Cary, NC).

## Results

The primary STURDY population consisted of 688 participants including 349 in the PHD group and 339 in the 200 IU/d dose group. In the burn-in cohort, 406 participants were randomized in each of three higher doses (*n* = 67) or the control dose (*n* = 205). A total of 687 participants had complete frailty assessment at baseline. The sample characteristics are displayed in Table 1 and a flow chart of the analytic sample is presented in Supplementary Fig. 1.

**Table 1:** Baseline characteristics by vitamin D treatment groups

	**All (*N* = 687)**	**Primary Analysis Population** **(*N* = 687)**		**Burn-in cohort (*n* = 405)**^b^			
		**Control (200 IU/d)** **(*n* = 339)**	**Pooled Higher Doses (PHD)**^**a**^** (*n *= 348)**	**200 IU/d** **(*n *= 205)**	**1000 IU/d** **(*n *= 66)**	**2000 IU/d** **(*n* = 67)**	**4000 IU/d** **(*n* = 67)**
**Age** (years), mean ± SD	77.1 ± 5.4	77.1 ± 5.4	77.2 ± 5.4	77.7 ± 5.6	76.4 ± 4.4	77.3 ± 4.6	79.1 ± 5.9
**Sex**, no. (%)							
Male	388 (56.5)	198 (58.4)	190 (54.6)	88 (42.9)	28 (42.4)	29 (43.3)	27 (40.3)
Female	299 (43.5)	141 (41.6)	158 (45.4)	117 (57.1)	38 (57.6)	38 (56.7)	40 (59.7)
**Race**, no. (%)^c^							
White	542 (79.7)	276 (82.4)	266 (77.1)	171 (83.4)	48 (72.7)	50 (75.8)	56 (83.6)
Black	124 (18.2)	55 (16.4)	69 (20.0)	32 (15.6)	13 (19.7)	15 (22.7)	10 (14.9)
Other	23 (3.4)	7 (2.1)	16 (4.6)	4 (2.0)	5 (7.6)	2 (3.0)	1 (1.5)
**BMI** (kg/m^2^), mean ± SD	30.5 ± 6.0	30.4 ± 6.3	30.6 ± 5.6	30.2 ± 6.3	31.5 ± 5.7	30.7 ± 6.4	30.3 ± 6.2
**Serum vitamin D** (ng/mL)^d^							
10 to 19, no. (%)	200 (29.1)	100 (29.5)	100 (28.7)	69 (33.7)	15 (22.7)	25 (37.3)	22 (32.8)
20 to 29, no. (%)	487 (70.9)	239 (70.5)	248 (71.3)	136 (66.3)	51 (77.3)	42 (62.7)	45 (67.2)
**Taking a personal vitamin D supplement**							
No. (%)	255 (37.1)	124 (36.6)	131 (37.6)	76 (37.1)	26 (39.4)	26 (38.8)	21 (31.3)
Median (IQR), IU/d	700 (600)	800 (586)	700 (600)	800 (586)	750 (500)	800 (500)	571 (400)
**Fell ≥ 1 time in prior year**, no. (%)	449 (65.4)	221 (65.2)	228 (65.5)	135 (65.9)	42 (63.6)	43 (64.2)	45 (67.2)
**Number of chronic conditions**^**e**^, mean ± SD	2.0 ± 1.2	1.9 ± 1.2	2.1 ± 1.2	2.0 ± 1.2	2.2 ± 1.3	2.1 ± 1.3	1.9 ± 1.2
**Frailty status**^f^, no. (%)							
Robust	208 (30.3)	105 (31.0)	103 (29.6)	60 (29.3)	19 (28.8)	25 (37.3)	23 (34.3)
Pre-frail	402 (58.5)	206 (60.8)	196 (56.3)	123 (60.0)	40 (60.6)	36 (53.7)	33 (49.3)
Frail	77 (11.2)	28 (8.2)	49 (14.1)	22 (10.7)	7 (10.6)	6 (9.0)	11 (16.4)

^a^Pooled Higher Doses denotes the combined 1000, 2000, and 4000 IU/d groups

^b^The four vitamin D groups were compared among participants in the burn-in cohort. The burn-in cohort from the dose-finding phase is an unbiased population for comparison of each higher dose versus control because these participants were randomized prior to the first adaptation of the randomization probabilities

^c^Race was self-reported by the participant from a list of 5 categories (American Indian or Alaska Native, Asian, Black or African American, Native Hawaiian or other Pacific Islander, White); more than one race could be reported by a participant

^d^The range of serum vitamin D level eligible for STURDY (10–29 ng/mL) includes levels termed deficient (< 20 ng/mL) or insufficient (20–29 ng/mL) by the Endocrine Society and overlaps with levels termed deficient (< 12 ng/mL), inadequate (12-19 ng/mL), or adequate (≥ 20) by the Institute of Medicine

^e^Chronic conditions included cardiovascular disease, hypertension, stroke, chronic lung disease, diabetes, kidney disease, liver disease, arthritis, Parkinson’s disease, and multiple sclerosis

^f^Frailty phenotype was defined as having three or more of the following condition: weight loss, exhaustion, slowness, low physical activity, and weakness

*IU/d* International units per day. *SD* Standard deviation. *BMI* Body mass index

**Fig. 1:**
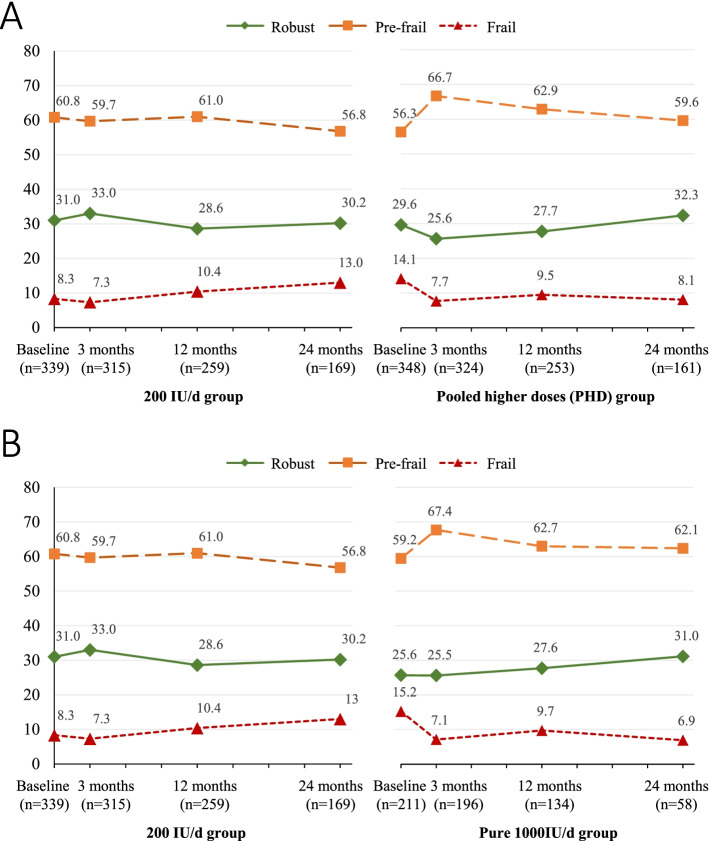
Percentage of participants with each frailty status at baseline and follow-up visits. **A** Pooled higher dose (PHD) vs. 200 IU/d. **B** Pure 1000 IU/d vs. 200 IU/d. PHD = Pooled higher doses (combined 1000 IU/d, 2000 IU/d, and 4000 IU/d). IU/d = International units per day

At baseline, 208 (30.3%) were robust, 402 (58.5%) were pre-frail, and 77 (11.2%) were frail (Table 1. The percentage of participants with each frailty status at follow-up visits are shown in Fig. 1 (PHD vs. control in Fig. 1A; pure 1000 IU/d vs. control in Fig. 1B) and Supplementary Table 1.

**Fig. 2:**
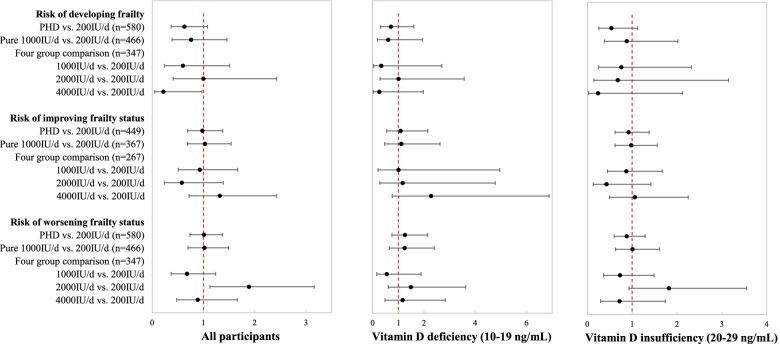
Hazard ratios (HRs) of developing frailty, improving frailty status, and worsening frailty status by vitamin D treatment groups in the confirmatory stage and dose-finding stage. The two sets of analyses comparing PHD vs. 200 IU/d and pure 1000 IU/d vs. 200 IU/d were conducted from the confirmatory stage. The four vitamin D treatment groups were compared among participants in the burn-in cohort from the dose-finding stage. This is an unbiased population for comparison of each higher dose versus control because these participants were randomized prior to the first adaptation of the randomization probabilities. PHD = pooled higher doses (combined 1000 IU/d, 2000 IU/d, and 4000 IU/d). IU/d = international units per day

Cox proportional hazard models showed no significant difference in risk of incident frailty (*n* = 580), improving frailty status (*n* = 449), or worsening frailty status (*n* = 580) comparing the PHD to the control dose (Fig. 2; Supplementary Table 2. However, for the analysis of the dose-finding stage comparing each higher dose to the control dose in the burn-in cohort, the 2000 IU/d dose group had nearly double the risk of worsening frailty status (hazard ratio (HR) = 1.89, 95% CI: 1.13–3.16, *p* = 0.015), while the 4000 IU/d dose had a lower risk of developing frailty during follow up (HR = 0.22, 95% CI: 0.05–0.97, *p* = 0.045) compared to the control dose (Supplementary Table 2. There were no significant associations between vitamin D doses and frailty status when stratifying by baseline serum 25(OH)D level (Supplementary Table 2).

GEE models showed no significant association between vitamin D treatment and frailty (Supplementary Table 3) in the primary PHD analysis (Model A; *n* = 656) and pure 1000 IU/d sensitivity analysis (Model B; *n* = 526). Analyses stratified by baseline serum 25(OH)D level showed no significant time by treatment interaction in the vitamin D deficient (10–19 ng/mL) group or the vitamin D insufficient (20–29 ng/mL) group (Supplementary Table 3, Model A and B).

When examining the five frailty components individually at baseline, 34 (5.1%) participants had weight loss, 77 (11.4%) had exhaustion, 184 (26.9%) had slow gait speed, 90 (13.1%) had low activity, and 392 (57.8%) had weakness. The frequency distribution of each frailty component by treatment group at each visit are shown in Supplementary Table 4. Over up to 24 months of follow up, Cox proportional hazards models showed no significant differences in risk of developing weight loss, exhaustion, low activity, or weakness between the PHD group and the control dose (Supplementary Table 5. Analyses stratified by baseline serum 25(OH)D level showed that, among participants with vitamin D insufficiency at baseline, the PHD group and pure 1000 IU/d group had a greater risk of developing slow gait speed compared to the control group (HR = 1.58, 95% CI: 1.01–2.47, *p* = 0.045; HR = 1.82, 95% CI: 1.10–3.02, *p* = 0.020, respectively). For four dose comparison in the burn-in cohort, participants with baseline vitamin D insufficiency in the 2000 IU/d group had a greater risk of slowness over time (HR = 2.24, 95% CI: 1.02–4.93, *p* = 0.045; Supplementary Table 5.

## Discussion

Our principal finding is that among older persons with low serum vitamin D level and at high risk for falling, high-dose vitamin D supplementation did not prevent frailty. This finding is consistent with the main STURDY findings which documented that vitamin D supplementation did not prevent falls or attenuate gait speed decline [16]. Although some analyses suggest that the 4000 IU/d dose might have beneficial effects on preventing frailty, this finding might be the results of a type 1 error.

To the best of our knowledge, our study is the first to examine the effects of high doses of vitamin D supplementation on frailty status using a randomized controlled trial approach. Previous studies were observational studies and only focused on the association between serum 25(OH)D levels and risk of frailty, not whether vitamin D supplementation can alter the risk of frailty over time [11, 13, 14, 20, 21]. For example, Buta and colleagues found that older women with < 10 ng/mL serum 25(OH)D level in the Women’s Health and Aging Study II had a three-fold increased risk of developing frailty over a mean period of 8.5 years, compared to those with ≥ 30 ng/mL of serum vitamin D level [13]. Only Bolzetta and colleagues have explored the association between low-dose daily vitamin D supplementation (≤ 600 IU/d) and risk of frailty over 8 years of follow up using observational data from the Osteoarthritis Initiative (OAI) database, with no significant associations found [15]. As observational studies, their findings are subject to residual confounding and biases.

There are several potential mechanisms that may underly the relationship between vitamin D and frailty. Muscle function may be one of the pathways linking vitamin D to frailty [22, 23]. A number of clinical trials have found that older adults undergoing vitamin D3 treatment, particularly with daily doses of 800 to1000IU, had increased lower extremity muscle strength [8, 9, 23, 24]. Another potential pathway from vitamin D to frailty might be fatigue [25, 26]. Although there was insufficient evidence pointing to the beneficial effects of vitamin D supplementation on preventing tiredness or fatigue in general populations [25–27], a randomized controlled trial (RCT) found that vitamin D treatment significantly reduced the risk of fatigue in adults with vitamin D deficiency [28]. However, we did not find similar significant associations between 1000 IU/d dose of vitamin D supplementation and frailty or its individual criteria among those with vitamin D deficiency. It is possible that vitamin D deficiency is a symptom of a state of physiologic dysregulation requiring more than supplementation to correct [29]. In addition, the small number of participants with vitamin D deficiency at baseline makes it difficult to disentangle the association between serum vitamin D and frailty and its individual criteria.

Several studies have raised the concern of the potential harmful effects of high vitamin D doses. Consistent with the STURDY main findings, where greater fall rates and fall-related factures were noted in the 2000 IU/d dose group [16, 30], our data showed an unfavorable effect of 2000 IU/d dose on risk of worsening frailty status. Some evidence suggests a U-shaped relationship between serum 25(OH)D level and risk of adverse health outcomes such as cardiovascular diseases [31–34]. A clinical trial reported decreased lower extremity muscle strength among community-dwelling postmenopausal women with low serum 25(OH)D level (< 50 nmol/L) taking 2800 IU/d of vitamin D3 supplementation compared to placebo [35]. Our study only recruited participants with low serum 25(OH)D level (< 30 ng/mL) at baseline. The main reasons for low vitamin D levels could be low dietary intake of vitamin D or lack of exposure to natural sunlight [29]. Other factors such as health conditions that affect absorption or metabolism of vitamin D or certain medication use may also contribute to this diminished state [29]. Further, these participants represent a group of older persons with low functioning and high risk for falls, who may likely remain at high risk of negative health outcomes regardless of vitamin D supplementation [36, 37].

Strengths of this study include high adherence and low attrition rates, a target older population with low serum 25(OH)D levels and high risk of falls, and enrollment of a diverse population. Our study also has several limitations. First, STURDY participants were allowed to take up to 1000 IU/d of supplemental vitamin D. However, all had low serum vitamin D levels at enrollment. Second, the target population is older adults with high risk for falls and low serum vitamin D levels; thus, the study findings may not be generalizable to other populations. Third, the control group received 200 IU/d of vitamin D rather than a placebo pill. Although this dose was selected to achieve ≥ 800 IU average total daily intake of vitamin D [16], it is uncertain whether 200 IU/d may influence frailty status compared to no supplementation. Fourth, fewer participants were assigned to the 2000 IU/d and 4000 IU/d groups due to the response-adaptive design, which may lead to reduced power to detect effects of these high doses of vitamin D supplementation on frailty status. Lastly, the RCT was not designed to assess frailty as an outcome. For some analyses (e.g., Cox proportional hazards models), small subsets of the original cohort were excluded based on frailty status at baseline. Thus, some results of this secondary data analysis should be considered with potential bias. In addition, we may not have sufficient power to detect statistically significant differences in HRs in sensitivity analyses and analyses with frailty components as the outcome. We also interpreted significant results with caution as these findings might be the results of type I error, given the small number of participants in each group and the small number of incident cases over follow up in the sensitivity analyses.

## Conclusions

Our study did not demonstrate a beneficial effect of vitamin D supplementation on frailty status. Although some analyses showed a reduced risk of frailty in the 4000 IU/d group, such results might be the result of type 1 error. Hence, replication of our findings is warranted.

## Supplementary Information

**Additional file 1** 

**Additional file 2 **

**Additional file 3** 

**Additional file 4 **

**Additional file 5** 

**Additional file 6 **

## Data Availability

The datasets generated and/or analyzed during the current study are not publicly available due to ethical reasons but are available from the corresponding author on reasonable request.

## Abbreviations

25(OH)D: 25-Hydroxyvitamin D
STURDY: Study To Understand Fall Reduction and Vitamin D in You
IU/d: International units per day
PHD: Pooled higher doses
CI: Confidence interval
HR: Hazard ratio
GEE: Generalized estimating equations
