# Supplementary figures and images for: The effects of vitamin D supplementation on frailty in older adults at risk for falls
Source: BMC Geriatr. 2022 Apr 10;22:312. doi: 10.1186/s12877-022-02888-w (PMC8994906; doi:10.1186/s12877-022-02888-w)

**Supplementary Figure 1. Flow chart of analytic sample.**

**
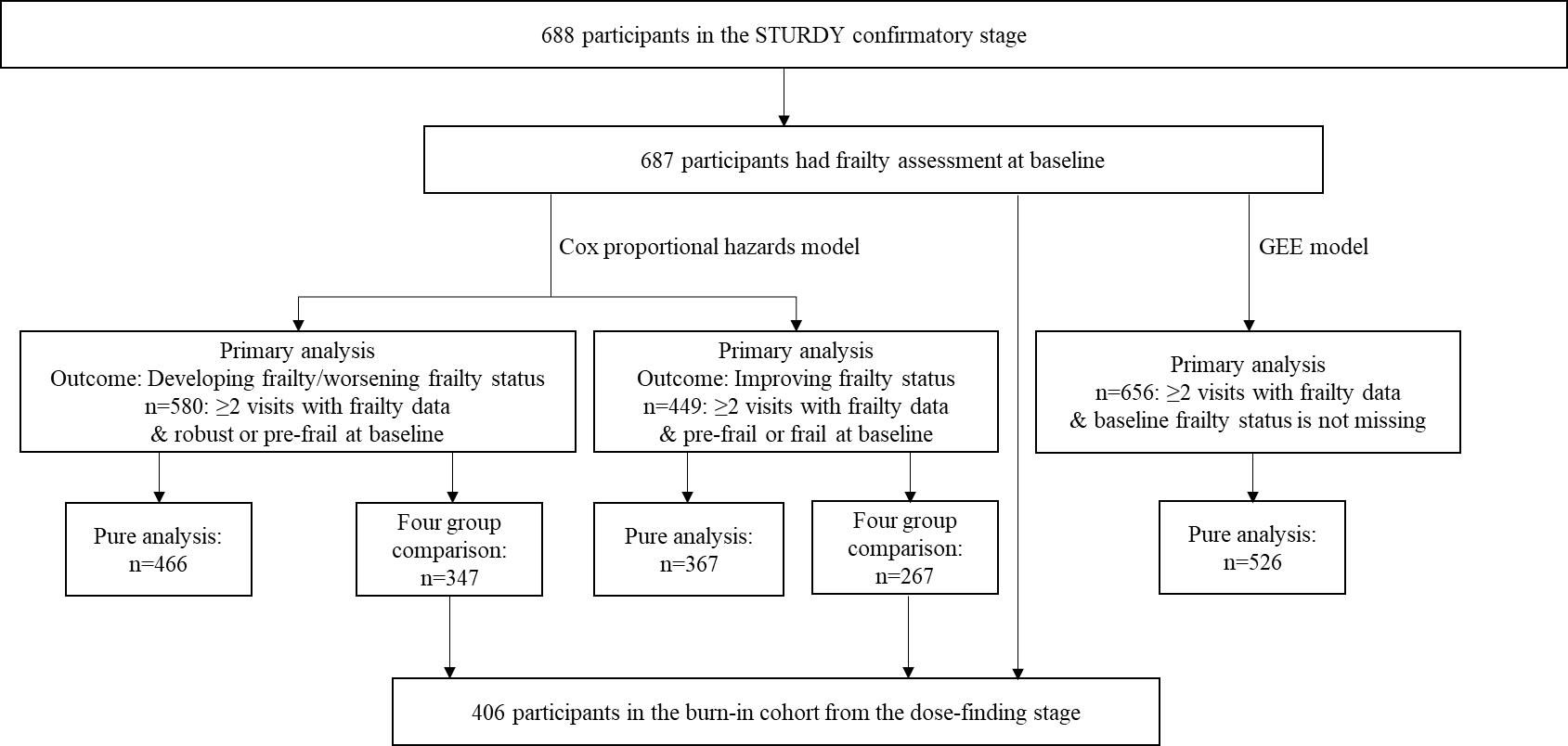
**

Supplement: Supplementary file 1 — Additional file 1 [file 12877_2022_2888_MOESM1_ESM.docx]
