# Supplementary material for: The effects of vitamin D supplementation on frailty in older adults at risk for falls
Source: BMC Geriatr. 2022 Apr 10;22:312. doi: 10.1186/s12877-022-02888-w (PMC8994906; doi:10.1186/s12877-022-02888-w)
Supplement: Supplementary file 2 — Additional file 2 [file 12877_2022_2888_MOESM2_ESM.docx]

**Supplementary Table 1. Frequency distribution [n (%)] of frailty status by vitamin D treatment group at baseline and follow-up visits in the confirmatory stage (Pooled higher doses (combined 1000IU/d, 2000IU/d, and 4000IU/d) group vs. 200IU/d dose group, and pure 1000IU/d vs. 200IU/d)**

|  | | **Baseline (n=687)** | | | **3 months (n=639)** | | | **12 months (n=512)** | | **24 months (n=330)** | |
| --- | --- | --- | --- | --- | --- | --- | --- | --- | --- | --- | --- |
|  | | 200IU/d  (n=339) | | PHD  (n=348) | 200IU/d  (n=315) | | PHD  (n=324) | 200IU/d  (n=259) | PHD  (n=253) | 200IU/d  (n=169) | PHD  (n=161) |
| **Robust** | | 105 (31.0) | | 103 (29.6) | 104 (33.0) | | 83 (25.6) | 74 (28.6) | 70 (27.7) | 51 (30.2) | 52 (32.3) |
| **Pre-frail** | | 206 (60.8) | | 196 (56.3) | 188 (59.7) | | 216 (66.7) | 158 (61.0) | 159 (62.9) | 96 (56.8) | 96 (59.6) |
| **Frail** | | 28 (8.2) | | 49 (14.1) | 23 (7.3) | | 25 (7.7) | 27 (10.4) | 24 (9.5) | 22 (13.0) | 13 (8.1) |
|  | **Baseline (n=550)** | | | | **3 months (n=511)** | | | **12 months (n=393)** | | **24 months (n=227)** | |
|  | 200IU/d (n=339) | | Pure 1000IU/d  (n=211) | | 200IU/d  (n=315) | Pure 1000IU/d  (n=196) | | 200IU/d  (n=259) | Pure 1000IU/d  (n=134) | 200IU/d  (n=169) | Pure 1000IU/d  (n=58) |
| **Robust** | 105 (31.0) | | 54 (25.6) | | 104 (33.0) | 50 (25.5) | | 74 (28.6) | 37 (27.6) | 51 (30.2) | 18 (31.0) |
| **Pre-frail** | 206 (60.8) | | 125 (59.2) | | 188 (59.7) | 132 (67.4) | | 158 (61.0) | 84 (62.7) | 96 (56.8) | 36 (62.1) |
| **Frail** | 28 (8.2) | | 32 (15.2)* | | 23 (7.3) | 14 (7.1) | | 27 (10.4) | 13 (9.7) | 22 (13.0) | 4 (6.9) |

*Note.* PHD=pooled higher doses. IU/d=international units per day.

**P*-value for Chi-square tests or Fisher’s exact tests < 0.05.
