# Supplementary material for: The effects of vitamin D supplementation on frailty in older adults at risk for falls
Source: BMC Geriatr. 2022 Apr 10;22:312. doi: 10.1186/s12877-022-02888-w (PMC8994906; doi:10.1186/s12877-022-02888-w)
Supplement: Supplementary file 3 — Additional file 3 [file 12877_2022_2888_MOESM3_ESM.docx]

**Supplementary Table 2. Cox proportional hazards models for the association between vitamin D dosage and frailty outcomes in the confirmatory stage and dose-finding stage**

|  | **No. of events** | **Average survival time (years)** | **All participants** | | **Stratified by baseline serum vitamin D level** | | | | | | |  |
| --- | --- | --- | --- | --- | --- | --- | --- | --- | --- | --- | --- | --- |
|  |  |  |  |  | **With vitamin D deficiency*** | |  | | **With vitamin D insufficiency*** | | | |
|  |  |  | **Hazard ratio (95% CI)** | **P value** | **Hazard ratio (95% CI)** | **P value** | |  | | **Hazard ratio (95% CI)** | **P value** | |
| **Risk of developing frailty**† |  |  |  |  |  |  | |  | |  |  | |
| PHD vs. 200IU/d (n=580) | 26/285 vs. 33/295 | 1.39 vs. 1.34 | 0.63 (0.37-1.08) | 0.091 | 0.71 (0.31-1.61) | 0.407 | |  | | 0.54 (0.26-1.12) | 0.098 | |
| Pure 1000IU/d vs. 200IU/d (n=466) | 13/171 vs. 33/295 | 1.13 vs. 1.34 | 0.76 (0.39-1.46) | 0.404 | 0.61 (0.19-1.94) | 0.404 | |  | | 0.88 (0.38-2.02) | 0.755 | |
| Four group comparison‡ |  |  |  |  |  |  | |  | |  |  | |
| 200IU/d (n=177) | 26 | 1.66 | ref | ref | ref | ref | |  | | ref | ref | |
| 1000IU/d (n=59) | 6 | 1.73 | 0.60 (0.24-1.51) | 0.154 | 0.34 (0.04-2.70) | 0.306 | |  | | 0.76 (0.25-2.32) | 0.635 | |
| 2000IU/d (n=57) | 8 | 1.04 | 1.00 (0.41-2.43) | 0.999 | 1.01 (0.29-3.57) | 0.986 | |  | | 0.68 (0.15-3.15) | 0.620 | |
| 4000IU/d (n=54) | 2 | 1.17 | 0.22 (0.05-0.97) | **0.045** | 0.26 (0.03-1.98) | 0.191 | |  | | 0.24 (0.03-2.12) | 0.201 | |
| **Risk of improving frailty status**§ |  |  |  |  |  |  | |  | |  |  | |
| PHD vs. 200IU/d **(**n=449) | 70/229 vs. 66/220 | 1.08 vs. 1.09 | 0.98 (0.69-1.38) | 0.890 | 1.09 (0.55-2.15) | 0.803 | |  | | 0.92 (0.62-1.38) | 0.692 | |
| Pure 1000IU/d vs. 200IU/d (n=367) | 42/147 vs. 66/220 | 0.85 vs. 1.09 | 1.03 (0.69-1.54) | 0.896 | 1.12 (0.47-2.63) | 0.800 | |  | | 0.98 (0.62-1.56) | 0.936 | |
| Four group comparison‡ |  |  |  |  |  |  | |  | |  |  | |
| 200IU/d (n=139) | 47 | 1.31 | ref | ref | ref | ref | |  | | ref | ref | |
| 1000IU/d (n=48) | 16 | 1.34 | 0.93 (0.51-1.67) | 0.800 | 1.01 (0.21-4.96) | 0.993 | |  | | 0.87 (0.45-1.67) | 0.675 | |
| 2000IU/d (n=39) | 6 | 0.97 | 0.58 (0.24-1.39) | 0.221 | 1.17 (0.28-4.78) | 0.833 | |  | | 0.43 (0.13-1.42) | 0.166 | |
| 4000IU/d (n=41) | 14 | 0.99 | 1.32 (0.72-2.43) | 0.370 | 2.28 (0.76-6.88) | 0.142 | |  | | 1.06 (0.50-2.25) | 0.872 | |
| **Risk of worsening frailty status**† |  |  |  |  |  |  | |  | |  |  | |
| PHD vs. 200IU/d (n=580) | 86/285 vs. 86/295 | 1.19 vs. 1.18 | 1.01 (0.74-1.37) | 0.952 | 1.26 (0.75-2.13) | 0.385 | |  | | 0.88 (0.60-1.29) | 0.508 | |
| Pure 1000IU/d vs. 200IU/d (n=466) | 44/171 vs. 86/295 | 1.02 vs. 1.18 | 1.02 (0.70-1.49) | 0.920 | 1.25 (0.65-2.40) | 0.509 | |  | | 1.01 (0.63-1.61) | 0.973 | |
| Four group comparison‡ |  |  |  |  |  |  | |  | |  |  | |
| 200IU/d (n=177) | 57 | 1.47 | ref | ref | ref | ref | |  | | ref | ref | |
| 1000IU/d (n=59) | 15 | 1.62 | 0.68 (0.37-1.24) | 0.204 | 0.55 (0.16-1.89) | 0.346 | |  | | 0.73 (0.36-1.49) | 0.386 | |
| 2000IU/d (n=57) | 23 | 0.88 | 1.89 (1.13-3.16) | **0.015** | 1.49 (0.61-3.63) | 0.382 | |  | | 1.82 (0.93-3.55) | 0.081 | |
| 4000IU/d (n=54) | 13 | 1.01 | 0.89 (0.48-1.66) | 0.715 | 1.17 (0.48-2.83) | 0.728 | |  | | 0.72 (0.30-1.74) | 0.465 | |

*Note.* PHD=pooled higher doses. IU/d=international units per day. CI=confidence interval.

Cox proportional hazard model adjusted for age, sex, race, comorbidities, body mass index, baseline serum vitamin D level, and history of falls. For analyses stratified by baseline serum vitamin D level, the models were adjusted for other covariates. Bolded p-values indicate statistically significant results (p<0.05).

*Vitamin D deficiency and insufficiency were defined as serum 25(OH)D level of 10-19ng/mL and 20-29ng/mL, respectively.

†Participants who were frail at baseline were removed for this analysis.

‡The four vitamin D groups were compared among participants in the burn-in cohort from the dose-finding stage. This is an unbiased population for comparison of each higher dose versus control because these participants were randomized prior to the first adaptation of the randomization probabilities.

§Participants who were robust at baseline were removed for this analysis.
