# Supplementary material for: The effects of vitamin D supplementation on frailty in older adults at risk for falls
Source: BMC Geriatr. 2022 Apr 10;22:312. doi: 10.1186/s12877-022-02888-w (PMC8994906; doi:10.1186/s12877-022-02888-w)
Supplement: Supplementary file 4 — Additional file 4 [file 12877_2022_2888_MOESM4_ESM.docx]

**Supplementary Table 3. Generalized estimating equations (GEE) models of changes in odds of frailty over time in the confirmatory stage**

|  | **All participants** | | | |  | **Stratified by baseline serum vitamin D level** | | | | | | | | | |
| --- | --- | --- | --- | --- | --- | --- | --- | --- | --- | --- | --- | --- | --- | --- | --- |
|  |  |  |  |  |  | **With vitamin D deficiency*** | | | | |  | **With vitamin D insufficiency*** | | | |
|  | B | SE | OR | p |  | B | SE | OR | p | |  | B | SE | OR | p |
| **Model A (primary analysis)**  **(PHD vs. 200IU/d)** | **(N=656)** | | | |  | **(n=188)** | | | |  | | **(n=468)** | | | |
| Time† | 0.40 | 0.18 | 1.49 | **0.030** |  | 0.32 | 0.24 | 1.38 | 0.190 | |  | 0.44 | 0.26 | 1.55 | 0.087 |
| Treatment |  |  |  |  |  |  |  |  |  | |  |  |  |  |  |
| 200IU/d | ref | ref | ref | ref |  | ref | ref | ref | ref | |  | ref | ref | ref | ref |
| PHD | -0.27 | 0.30 | 0.76 | 0.356 |  | -0.65 | 0.52 | 0.52 | 0.214 | |  | -0.08 | 0.37 | 0.92 | 0.827 |
| Treatment * time |  |  |  |  |  |  |  |  |  | |  |  |  |  |  |
| 200IU/d | ref | ref | ref | ref |  | ref | ref | ref | ref | |  | ref | ref | ref | ref |
| PHD | -0.14 | 0.27 | 0.87 | 0.613 |  | 0.27 | 0.42 | 1.31 | 0.527 | |  | -0.37 | 0.36 | 0.69 | 0.303 |
| **Model B**  **(pure 1000IU/d vs. 200IU/d)** | **(N=526)** | | | |  | **(n=143)** | | | |  | | **(n=383)** | | | |
| Time† | 0.40 | 0.18 | 1.49 | **0.024** |  | 0.44 | 0.25 | 1.56 | 0.078 | |  | 0.42 | 0.25 | 1.52 | 0.099 |
| Treatment |  |  |  |  |  |  |  |  |  | |  |  |  |  |  |
| 200IU/d | ref | ref | ref | ref |  | ref | ref | ref | ref | |  | ref | ref | ref | ref |
| Pure 1000IU/d | -0.18 | 0.32 | 0.83 | 0.568 |  | -0.23 | 0.58 | 0.79 | 0.685 | |  | -0.21 | 0.40 | 0.81 | 0.610 |
| Treatment * time |  |  |  |  |  |  |  |  |  | |  |  |  |  |  |
| 200IU/d | ref | ref | ref | ref |  | ref | ref | ref | ref | |  | ref | ref | ref | ref |
| Pure 1000IU/d | -0.25 | 0.33 | 0.78 | 0.435 |  | -0.38 | 0.40 | 0.69 | 0.343 | |  | 0.02 | 0.42 | 1.02 | 0.963 |

*Note.* PHD=pooled higher doses. IU/d=international units per day. OR=odds ratio.

Generalized estimating equation (GEE) model adjusted for age, sex, race, body mass index, comorbidities, baseline serum vitamin D level, history of falls, and baseline frailty status. For analyses stratified by baseline serum vitamin D level, the models were adjusted for other covariates. Bolded p-values indicate statistically significant results (p<0.05).

* Vitamin D deficiency and insufficiency were defined as serum 25(OH)D level of 10-19ng/mL and 20-29ng/mL, respectively.

†Time was centered at 3 months.
