# Supplementary material for: The effects of vitamin D supplementation on frailty in older adults at risk for falls
Source: BMC Geriatr. 2022 Apr 10;22:312. doi: 10.1186/s12877-022-02888-w (PMC8994906; doi:10.1186/s12877-022-02888-w)
Supplement: Supplementary file 5 — Additional file 5 [file 12877_2022_2888_MOESM5_ESM.docx]

**Supplementary Table 4. Frequency distribution [n (%)] of frailty components by vitamin D treatment groups at baseline and follow-up visits in the confirmatory stage**

|  | **Baseline (n=688)** | | **3 months (n=642)** | | **12 months (n=521)** | | **24 months (n=339)** | |
| --- | --- | --- | --- | --- | --- | --- | --- | --- |
|  | 200IU/d  (n=339) | PHD  (n=349) | 200IU/d  (n=317) | PHD  (n=325) | 200IU/d  (n=262) | PHD  (n=259) | 200IU/d  (n=174) | PHD  (n=165) |
| **Weight loss** | 16 (4.8) | 18 (5.3) | 6 (1.9) | 6 (1.9) | 16 (6.1) | 12 (4.7) | 9 (5.3) | 10 (6.2) |
| Missingness | 8 | 7 | 2 | 1 | 1 | 6 | 4 | 4 |
| **Exhaustion** | 26 (7.8) | 51 (14.8)* | 25 (7.9) | 32 (9.9) | 30 (11.5) | 28 (10.8) | 18 (10.3) | 11 (6.8) |
| Missingness | 7 | 5 | 1 | 1 | 1 | 0 | 0 | 3 |
| **Slowness** | 79 (23.4) | 105 (30.3)* | 71 (22.9) | 98 (30.6)* | 69 (26.7) | 77 (31.6) | 46 (28.4) | 52 (34.2) |
| Missingness | 1 | 2 | 7 | 5 | 4 | 16 | 12 | 13 |
| **Low activity** | 47 (13.9) | 43 (12.4) | 33 (10.5) | 42 (13.2) | 34 (13.2) | 31 (12.4) | 25 (15.0) | 19 (12.0) |
| Missingness | 0 | 2 | 4 | 6 | 5 | 8 | 7 | 6 |
| **Weakness** | 192 (57.3) | 200 (58.3) | 187 (60.9) | 198 (62.7) | 147 (58.3) | 154 (62.6) | 100 (61.4) | 88 (57.9) |
| Missingness | 4 | 6 | 10 | 9 | 10 | 13 | 11 | 13 |
|  | **Baseline (n=551)** | | **3 months (n=513)** | | **12 months (n=402)** | | **24 months (n=233)** | |
|  | 200IU/d  (n=339) | Pure 1000IU/d  (n=212) | 200IU/d  (n=317) | Pure 1000IU/d  (n=196) | 200IU/d  (n=262) | Pure 1000IU/d  (n=140) | 200IU/d  (n=174) | Pure 1000IU/d  (n=59) |
| **Weight loss** | 16 (4.8) | 13 (6.3) | 6 (1.9) | 3 (1.5) | 16 (6.1) | 9 (6.7) | 9 (5.3) | 3 (5.2) |
| Missingness | 8 | 4 | 2 | 0 | 1 | 6 | 4 | 1 |
| **Exhaustion** | 26 (7.8) | 33 (15.8)* | 25 (7.9) | 19 (9.7) | 30 (11.5) | 16 (11.4) | 18 (10.3) | 2 (3.4) |
| Missingness | 7 | 3 | 1 | 0 | 1 | 0 | 0 | 0 |
| **Slowness** | 79 (23.4) | 63 (30.0) | 71 (22.9) | 63 (32.3)* | 69 (26.7) | 39 (29.8) | 46 (28.4) | 15 (27.3) |
| Missingness | 1 | 2 | 7 | 1 | 4 | 9 | 12 | 4 |
| **Low activity** | 47 (13.9) | 27 (12.9) | 33 (10.5) | 25 (13.0) | 34 (13.2) | 16 (12.1) | 25 (15.0) | 7 (12.3) |
| Missingness | 0 | 2 | 4 | 4 | 5 | 8 | 7 | 2 |
| **Weakness** | 192 (57.3) | 130 (63.1) | 187 (60.9) | 123 (65.1) | 147 (58.3) | 80 (61.5) | 100 (61.4) | 31 (56.4) |
| Missingness | 4 | 6 | 10 | 7 | 10 | 10 | 11 | 4 |

*Note.* PHD=pooled higher doses. IU/d=international units per day.

**P*-value for Chi-square tests or Fisher’s exact tests < 0.05.
