# Supplementary material for: The effects of vitamin D supplementation on frailty in older adults at risk for falls
Source: BMC Geriatr. 2022 Apr 10;22:312. doi: 10.1186/s12877-022-02888-w (PMC8994906; doi:10.1186/s12877-022-02888-w)
Supplement: Supplementary file 6 — Additional file 6 [file 12877_2022_2888_MOESM6_ESM.docx]

**Supplementary Table 5. Cox proportional hazards model for the association between vitamin D dosage and frailty components development in the confirmatory stage and dose-finding stage**

|  | **No. of events** | **Average survival time (years)** | **All participants** | |  | **Stratified by baseline serum vitamin D level** | | | | |
| --- | --- | --- | --- | --- | --- | --- | --- | --- | --- | --- |
|  |  |  |  |  |  | **With vitamin D deficiency*** | |  | **With vitamin D insufficiency*** | |
|  |  |  | **Hazard ratio (95% CI)** | **P-value** |  | **Hazard ratio (95% CI)** | **P-value** |  | **Hazard ratio (95% CI)** | **P-value** |
| **Weight loss** |  |  |  |  |  |  |  |  |  |  |
| PHD vs. 200IU/d (n=627) | 26/318 vs. 21/309 | 1.38 vs. 1.39 | 1.01 (0.56-1.82) | 0.977 |  | 1.54 (0.57-4.16) | 0.397 |  | 0.87 (0.40-1.90) | 0.729 |
| Pure 1000IU/d vs. 200IU/d (n=502) | 15/193 vs. 21/309 | 1.12 vs. 1.39 | 1.41 (0.70-2.86) | 0.338 |  | 1.10 (0.22-5.44) | 0.911 |  | 1.78 (0.76-4.21) | 0.188 |
| Four group comparison* |  |  |  |  |  |  |  |  |  |  |
| 200IU/d (n=188) | 19 | 1.71 | ref | ref |  | ref | ref |  | ref | ref |
| 1000IU/d (n=65) | 5 | 1.74 | 0.71 (0.25-1.98) | 0.508 |  | 0.53 (0.05-5.58) | 0.595 |  | 0.75 (0.23-2.44) | 0.635 |
| 2000IU/d (n=61) | 6 | 1.06 | 1.52 (0.57-4.03) | 0.405 |  | 6.27 (1.07-36.79) | **0.042** |  | 0.91 (0.24-3.53) | 0.894 |
| 4000IU/d (n=61) | 1 | 1.12 | 0.22 (0.03-1.65) | 0.140 |  | 0.56 (0.06-4.99) | 0.605 |  | -- | -- |
| **Exhaustion** |  |  |  |  |  |  |  |  |  |  |
| PHD vs. 200IU/d (n=582) | 31/283 vs. 45/299 | 1.35 vs. 1.33 | 0.65 (0.41-1.04) | 0.073 |  | 0.58 (0.25-1.31) | 0.190 |  | 0.67 (0.37-1.19) | 0.174 |
| Pure 1000IU/d vs. 200IU/d (n=470) | 14/171 vs. 45/299 | 1.12 vs. 1.33 | 0.55 (0.29-1.03) | 0.062 |  | 0.42 (0.12-1.44) | 0.167 |  | 0.56 (0.26-1.18) | 0.127 |
| Four group comparison* |  |  |  |  |  |  |  |  |  |  |
| 200IU/d (n=180) | 32 | 1.65 | ref | ref |  | ref | ref |  | ref | ref |
| 1000IU/d (n=58) | 5 | 1.74 | 0.38 (0.15-1.02) | 0.054 |  | 0.26 (0.03-2.02) | 0.198 |  | 0.36 (0.10-1.25) | 0.108 |
| 2000IU/d (n=56) | 8 | 1.05 | 1.10 (0.49-2.45) | 0.822 |  | 0.99 (0.27-3.64) | 0.990 |  | 1.41 (0.51-3.88) | 0.507 |
| 4000IU/d (n=53) | 7 | 1.11 | 1.02 (0.44-2.35) | 0.965 |  | 0.82 (0.23-2.93) | 0.759 |  | 1.21 (0.40-3.68) | 0.735 |
| **Slow gait speed** |  |  |  |  |  |  |  |  |  |  |
| PHD vs. 200IU/d (n=476) | 66/231 vs. 53/245 | 1.20 vs. 1.29 | 1.29 (0.90-1.86) | 0.168 |  | 0.75 (0.38-1.47) | 0.399 |  | 1.58 (1.01-2.47) | **0.045** |
| Pure 1000IU/d vs. 200IU/d (n=385) | 36/140 vs. 53/245 | 1.01 vs. 1.29 | 1.41 (0.92-2.17) | 0.115 |  | 0.70 (0.27-1.78) | 0.449 |  | 1.82 (1.10-3.02) | **0.020** |
| Four group comparison* |  |  |  |  |  |  |  |  |  |  |
| 200IU/d (n=155) | 39 | 1.55 | ref | ref |  | ref | ref |  | ref | ref |
| 1000IU/d (n=41)† | 8 | 1.58 | 0.70 (0.33-1.51) | 0.364 |  | - | - |  | 1.06 (0.47-2.38) | 0.885 |
| 2000IU/d (n=41) | 13 | 0.92 | 1.83 (0.95-3.50) | 0.069 |  | 0.80 (0.20-3.19) | 0.751 |  | 2.24 (1.02-4.93) | **0.045** |
| 4000IU/d (n=47) | 11 | 1.04 | 1.20 (0.60-2.40) | 0.616 |  | 0.66 (0.18-2.39) | 0.529 |  | 1.50 (0.65-3.47) | 0.338 |
| **Low activity** |  |  |  |  |  |  |  |  |  |  |
| PHD vs. 200IU/d (n=571) | 44/293 vs. 32/278 | 1.33 vs. 1.32 | 1.05 (0.66-1.67) | 0.845 |  | 0.67 (0.31-1.48) | 0.324 |  | 1.36 (0.74-2.52) | 0.326 |
| Pure 1000IU/d vs. 200IU/d (n=455) | 23/177 vs. 32/278 | 1.09 vs. 1.32 | 1.21 (0.69-2.13) | 0.504 |  | 0.92 (0.34-2.48) | 0.866 |  | 1.56 (0.76-3.18) | 0.224 |
| Four group comparison* |  |  |  |  |  |  |  |  |  |  |
| 200IU/d (n=161) | 21 | 1.65 | ref | ref |  | ref | ref |  | ref | ref |
| 1000IU/d (n=59) | 8 | 1.66 | 0.80 (0.32-1.98) | 0.628 |  | 0.79 (0.17-3.69) | 0.759 |  | 0.96 (0.30-3.07) | 0.944 |
| 2000IU/d (n=57) | 13 | 0.96 | 2.10 (0.98-4.49) | 0.057 |  | 1.79 (0.50-6.32) | 0.369 |  | 2.45 (0.88-6.84) | 0.086 |
| 4000IU/d (n=56) | 3 | 1.12 | 0.39 (0.12-1.35) | 0.138 |  | 0.26 (0.03-2.20) | 0.217 |  | 0.50 (0.11-2.36) | 0.382 |
| **Weakness** |  |  |  |  |  |  |  |  |  |  |
| PHD vs. 200IU/d (n=287) | 70/144 vs. 65/143 | 1.11 vs. 1.10 | 0.98 (0.69-1.41) | 0.927 |  | 1.30 (0.62-2.73) | 0.493 |  | 0.92 (0.60-1.40) | 0.688 |
| Pure 1000IU/d vs. 200IU/d (n=224) | 35/81 vs. 65/143 | 0.99 vs. 1.10 | 0.90 (0.57-1.41) | 0.640 |  | 1.00 (0.39-2.55) | 0.998 |  | 0.90 (0.53-1.52) | 0.687 |
| Four group comparison* |  |  |  |  |  |  |  |  |  |  |
| 200IU/d (n=86) | 39 | 1.35 | ref | ref |  | ref | ref |  | ref | ref |
| 1000IU/d (n=29) | 14 | 1.41 | 0.70 (0.36-1.38) | 0.305 |  | 0.17 (0.02-1.53) | 0.114 |  | 0.92 (0.43-1.94) | 0.820 |
| 2000IU/d (n=33) | 15 | 1.25 | 1.30 (0.70-2.40) | 0.407 |  | 2.91 (0.73-11.62) | 0.130 |  | 1.21 (0.59-2.46) | 0.604 |
| 4000IU/d (n=29) | 14 | 1.35 | 1.61 (0.84-3.08) | 0.149 |  | 2.26 (0.75-6.78) | 0.146 |  | 1.56 (0.63-3.87) | 0.338 |

*Note.* PHD=pooled higher doses. IU/d=international units per day. CI=confidence interval.

Cox proportional hazard model adjusted for age, sex, comorbidities, body mass index, baseline serum vitamin D level, and history of falls. For analyses stratified by baseline serum vitamin D level, the models were adjusted for other covariates. Bolded p-values indicate statistically significant results (p<0.05).

*The four vitamin D groups were compared among participants in the burn-in cohort from the dose-finding stage. This is an unbiased population for comparison of each higher dose versus control because these participants were randomized prior to the first adaptation of the randomization probabilities.

† No events in the 1000IU/d dose group among participants with vitamin D deficiency at baseline.
